# Wearing a KN95/FFP2 facemask has no measureable effect on functional activity in a challenging working memory n-back task

**DOI:** 10.3389/fnhum.2024.1374625

**Published:** 2024-05-06

**Authors:** Marie-Louise Montandon, Sven Haller, Cristelle Rodriguez, François R. Herrmann, Panteleimon Giannakopoulos

**Affiliations:** ^1^Memory Center, Department of Rehabilitation and Geriatrics, Geneva University Hospitals, Geneva, Switzerland; ^2^CIMC—Centre d’Imagerie Médicale de Cornavin, Geneva, Switzerland; ^3^Department of Surgical Sciences, Radiology, Uppsala University, Uppsala, Sweden; ^4^Faculty of Medicine of the University of Geneva, Geneva, Switzerland; ^5^Department of Radiology, Beijing Tiantan Hospital, Capital Medical University, Beijing, China; ^6^Division of Institutional Measures, Medical Direction, Geneva University Hospitals, Geneva, Switzerland; ^7^Department of Rehabilitation and Geriatrics, Geneva University Hospitals and University of Geneva, Geneva, Switzerland; ^8^Department of Psychiatry, Faculty of Medicine, University of Geneva, Geneva, Switzerland

**Keywords:** facemask, working memory, fMRI, independent component analysis, functional connectivity

## Abstract

**Introduction:**

Wide use of facemasks is one of the many consequences of the COVID-19 pandemic.

**Methods:**

We used an established working memory n-back task in functional magnetic resonance imaging (fMRI) to explore whether wearing a KN95/FFP2 facemask affects overall performance and brain activation patterns. We provide here a prospective crossover design 3 T fMRI study with/without wearing a tight FFP2/KN95 facemask, including 24 community-dwelling male healthy control participants (mean age ± SD = 37.6 ± 12.7 years) performing a 2-back task. Data analysis was performed using the FSL toolbox, performing both task-related and functional connectivity independent component analyses.

**Results:**

Wearing an FFP2/KN95 facemask did not impact behavioral measures of the 2-back task (response time and number of errors). The 2-back task resulted in typical activations in working-memory related areas in both MASK and NOMASK conditions. There were no statistically significant differences in MASK versus NOMASK while performing the 2-back task in both task-related and functional connectivity fMRI analyses.

**Conclusion:**

The effect of wearing a tight FFP2/KN95 facemasks did not significantly affect working memory performance and brain activation patterns of functional connectivity.

## 1 Introduction

Wearing a facemask in professional settings is a usual procedure mainly for surgical purposes. The COVID-19 pandemic rendered the long-term use of facemasks a daily reality for the vast majority of citizens. This was the case in professional and even private interactions but also in MRI scanning facilities. This practice was widely accepted and well tolerated to limit the propagation of the virus mainly in vulnerable groups. Under normal conditions and mild exercise, wearing a facemask does not affect sensorial processing, motor or high-level cognitive performances (Morris et al., 2020; Slimani et al., 2021; Wells et al., 2023). However, some lines of evidence indicated that facemasks may alter concentration and visual attention in maximal running aerobic tests (Slimani et al., 2021). By impacting social interaction, facemasks modify the neural responses to recognition of facial cues but also pivotal human abilities serving our daily behavior such as emotion recognition that stimulate empathic responses, perceived closeness, trust attribution and even re-identification of unmasked faces (Ferrari et al., 2021; Grundmann et al., 2021; Marini et al., 2021, 2022; Proverbio and Cerri, 2022; Tsantani et al., 2022; Proverbio et al., 2023). In contrast to behavioral changes, data on the effect of wearing a facemask on brain activation remain rare. Two previous studies indicated that this practice has a subtle but still significant effect on cerebral blood flow and oxygen saturation but also BOLD (blood oxygenation level dependent) baseline signals. This BOLD effect is the basis of functional MRI. In short, local neuronal activity is associated with increased demands on oxygen and associated with a local vascular response which in turn modifies the local concentration ratio of oxygenated versus deoxygenated hemoglobin. This modifies the local magnetic properties that can be assessed using a dedicated MRI pulse sequence. The BOLD response is therefore an indirect vascular response that can be measured in fMRI. In a previous study, we also reported a small yet significant alteration in at rest functional connectivity limited to higher-level salience network in an independent sample of community-dwelling healthy controls (Haller et al., 2022). Functional connectivity fMRI is based on the principle assumption that if spontaneous fluctuations in the BOLD signal in two regions are not random but correlated, then these two regions are probably functionally connected. The more similar the BOLD signal fluctuations over time in two regions, i.e., the higher the correlation between two regions, the more likely is the functional connectivity between them. This study included only at rest fMRI analysis and cannot thus define whether brain activation during highly demanding cognitive tasks may be affected by wearing a facemask. To address this issue, we performed a cross-sectional study comparing the behavioral and fMRI patterns when wearing or not a tight KN95/FFP2 facemask during the performance of a 2-back working memory task in healthy controls. The 2-back working memory task is well established for fMRI studies and previously used by our group (Haller et al., 2013, 2014; Sinanaj et al., 2015; Haller et al., 2017; Zanchi et al., 2017). Since we anticipated only subtle effects related to the wearing of a facemask, the selection of the highly demanding 2-back condition, that implies the mobilization of working memory and not only attentional resources, allowed for obtaining the strong and reproducible fMRI activations needed for the detection of small group differences.

## 2 Materials and methods

### 2.1 Participants

This prospective study was approved by the Ethics Committee of the University Hospitals and University of Geneva, Switzerland, the study was in accordance with the Declaration of Helsinki, and all participants gave written informed consent. The study included 24 community-dwelling male healthy control participants (mean age ± SD = 37.6 ± 12.7 years) recruited via advertisements in local media. The following exclusion criteria were applied: a. presence or history of a chronic psychiatric disorder (psychosis, bipolar disorder) b. history of loss of consciousness lasting longer than 30 min, c. history of head injury or post-concussion symptoms, d. history of auditory or visual deficits, seizure and neurological disorders, and e. regular use of psychotropic medications and alcohol. The exclusion of acute psychiatric disorders was confirmed by the Mini Neuropsychiatric Interview (Stanga et al., 2019).

### 2.2 Working memory task

We used a classical fMRI experiment in order to explore the presence of subtle differences in the spatial distribution of brain activation between MASK and NOMASK conditions. The fMRI technic was selected since it makes possible to examine the functional reactivity of the human brain facing increased cognitive demands and in particular working memory activation. Previous contributions established in detail the patterns of brain activation during the successful performance of the 2-back task both in controls and clinical populations (Haller et al., 2013, 2014; Sinanaj et al., 2015; Haller et al., 2017; Zanchi et al., 2017). Briefly, a sequence of letters was presented visually on an MR compatible canvas in the MRI scanner. In the active 2-back condition (high WM demand), targets are letters that are identical to the letter presented two items ago (e.g., “a f h f”). In the control condition 0-back (visual processing with minimal WM requirements), the target is a pre-defined letter (e.g., “x”). Both conditions are contrasted to evaluate the effect of WM demand in 2-back versus the control condition 0-back.

**Figure 1:**
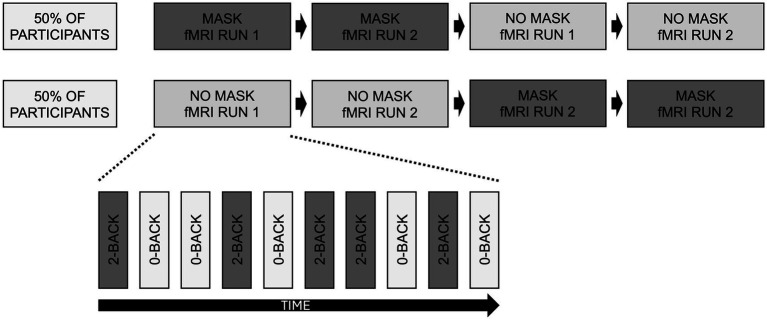
Schematic illustration of the experimental setup. In a cross-over design, half of participants first performed 2 fMRI runs MASK and then 2 fMRI runs NO MASK, while the other half of participants performed the opposite order. Each run consisted of 10 blocks, 5 blocks of 0-back and 5 blocks of 2-backs in pseudo-randomised order.

For the fMRI experiment, we used a cross-over design: half of the participants had first MASK then NOMASK condition, the other half had the inverse order. All participants had the same KN95/FFP2 facemask (a commercial model without metal to be compatible with the MR scanning). First, participants were familiarized with the task demands outside the MRI using a training session. The actual fMRI protocol consisted of four runs (2 × MASK and 2 × NO MASK). Each run included alternation blocks of 35 s each for conditions 2-back and 0-back with interleaving rest conditions of 15 s to allow the hemodynamic response to recover from the previous block. Each n-back condition (0-back or 2-back) was repeated five times in a pseudo-randomized order. Participants provided response (target versus no target) via an MR compatible response box for targets (33% of trials) and another button for non-targets (Figure 1).

The entire MRI scanning lasted approximatively 1 h. We made sure that participants had the facemask on for 10 min before the start of MASK condition, and no facemask for 10 min before NOMASK condition. To avoid potential bias of the resting fMRI results due to basic physiologic parameters, we monitored breathing and heart rate during the fMRI runs.

### 2.3 MR imaging

MR images were acquired using a 3 T MRI scanner (MAGNETOM PRISMA, Siemens) at Campus Biotech Geneva^1^. MR sequence parameters are listed in Table 1. Functional echo-planar imaging had the following essential parameters: 66 slices, slice thickness = 2.0 mm, voxel size = 2.0 mm × 2.0 mm × 2.0 mm, repetition time = 1,000 ms, echo time = 32 ms, flip angle = 50°, and field of view = 224 mm, resulting in 8.22 min per fMRI run. Each participant performed 2 runs in a crossover design, once with and once without an FFP2/KN 95 facemask. An additionally acquired 3DT1 sequence (208 slices, slice thickness = 1.0 mm; voxel size = 1 × 1 × 1 mm; repetition time = 2,300 ms; echo time = 2.26 ms; flip angle = 8°; field of view = 256 mm) was used for spatial normalization and registration.

**Table 1:** MR sequence parameters.

MR sequence parameters	
Sequence parameters of the resting-state fMRI protocol	
Number of slices	66
Slice thickness (mm)	2.0
Voxel size (mm^2^)	2.0 × 2.0 × 2.0
Repetition time (ms)	1,000
Echo time (ms)	32
Flip angle (°)	50
Field of view (mm)	224
Acquisition time (min)	8:22
3DT1 sequence parameters	
Number of slices	208
Slice thickness (mm)	1.0
Voxel size (mm^2^)	1.0 × 1.0 × 1.0
Repetition time (ms)	2,300
Echo time (ms)	2.26
Flip angle (°)	8
Field of view (mm)	256
Acquisition time (min)	4:44

## 3 Statistical analysis

### 3.1 Behavioral measures statistical analysis

The behavioral measures, notably reaction time and number of errors, were analyzed using Graphpad Prism Version 9^2^ using repeated measures parametric *t*-test for MASK versus NOMASK without correction for multiple comparisons (to make sure that eventual small changes are not masked by multiple comparison corrections).

### 3.2 Image analysis

#### 3.2.1 Task-related fMRI analysis

Task related fMRI analysis was performed in FSL version 5.0.10 using the standard processing pipeline FEAT as described in detail (Jenkinson et al., 2012), *equivalent to previous analyses* (Haller et al., 2014; Sinanaj et al., 2015; Haller et al., 2017; Zanchi et al., 2017).

The main contrast of interest was MASK versus NOMASK, which was analyses for 2-back only, 0-back only and 2-back versus 0-back. AGE and gender were used as non-explanatory co-regressors. A statistical threshold was defined as corrected *p* < 0.05 using the false discovery rate (FDR) (Genovese et al., 2002).

#### 3.2.2 Resting fMRI analysis

Resting fMRI analysis was performed in FSL version 6.0.6.1 using the standard processing pipeline MELODIC as described in detail (Jenkinson et al., 2012). First, a tensorial independent component analysis (TICA) was performed using 20 independent components. Then, the s-modes, a unitless measure of the activations strength of each component, was compared for MASK versus NOMASK using parametric tests. Finally, a dual regression analysis was performed using the same setup as above, i.e., first for 2-back only, then 0-back only and 2-back versus 0-back. Again, age and gender were used as non-explanatory co-regressors with a statistical threshold of corrected *p* < 0.05 FDR (Genovese et al., 2002).

## 4 Results

### 4.1 Behavioral data

There was no different in the behavioral data, notably reaction time and number of errors for MASK versus NOMASK during both conditions 0-back and 2-back (Table 2).

**Table 2:** Analysis of behavioral measures notably reaction time (RT) and number of errors (ERR) for the conditions 0-back and 2-back.

	MASK	NOMASK	STATS
0-back RT	439.6 ± 71.7 ms	435.7 ± 80.4 ms	NS
2-back RT	649.3 ± 172.5 ms	631 ± 190.6 ms	NS
0-back ERR	61 ± 12.1	87 ± 17	NS
2-back ERR	446 ± 85.8	433 ± 83.2	NS

No significant differences were found in equivalent conditions for MASK versus NOMASK (STATS, NS, non-significant).

### 4.2 Task-related activation

**Figure 2:**
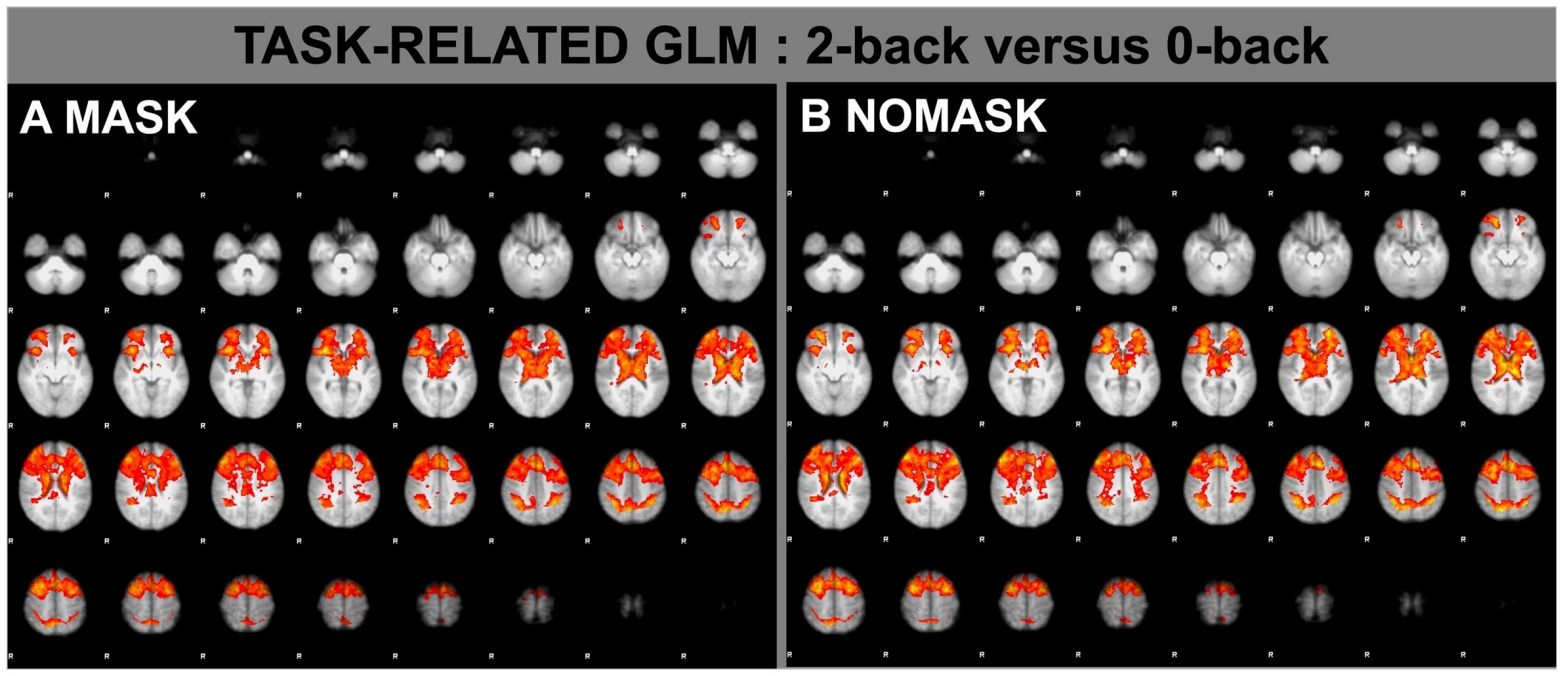
Task-related fMRI analysis (FEAT) for the contrast of 2-back versus 0-back while wearing a mask **(A)** and without wearing a mask **(B)**. *p* < 0.05 corrected FDR.

The main effect of 2-back versus 0-back resulted in the typical and well-established working-memory pattern of activations for both only MASK and only NOMASK (Figure 2). The direct comparison of MASK versus NOMASK as well as NOMASK versus MASK yielded no supra-threshold activations.

### 4.3 Resting fMRI activation (TICA and dual regression)

**Figure 3:**
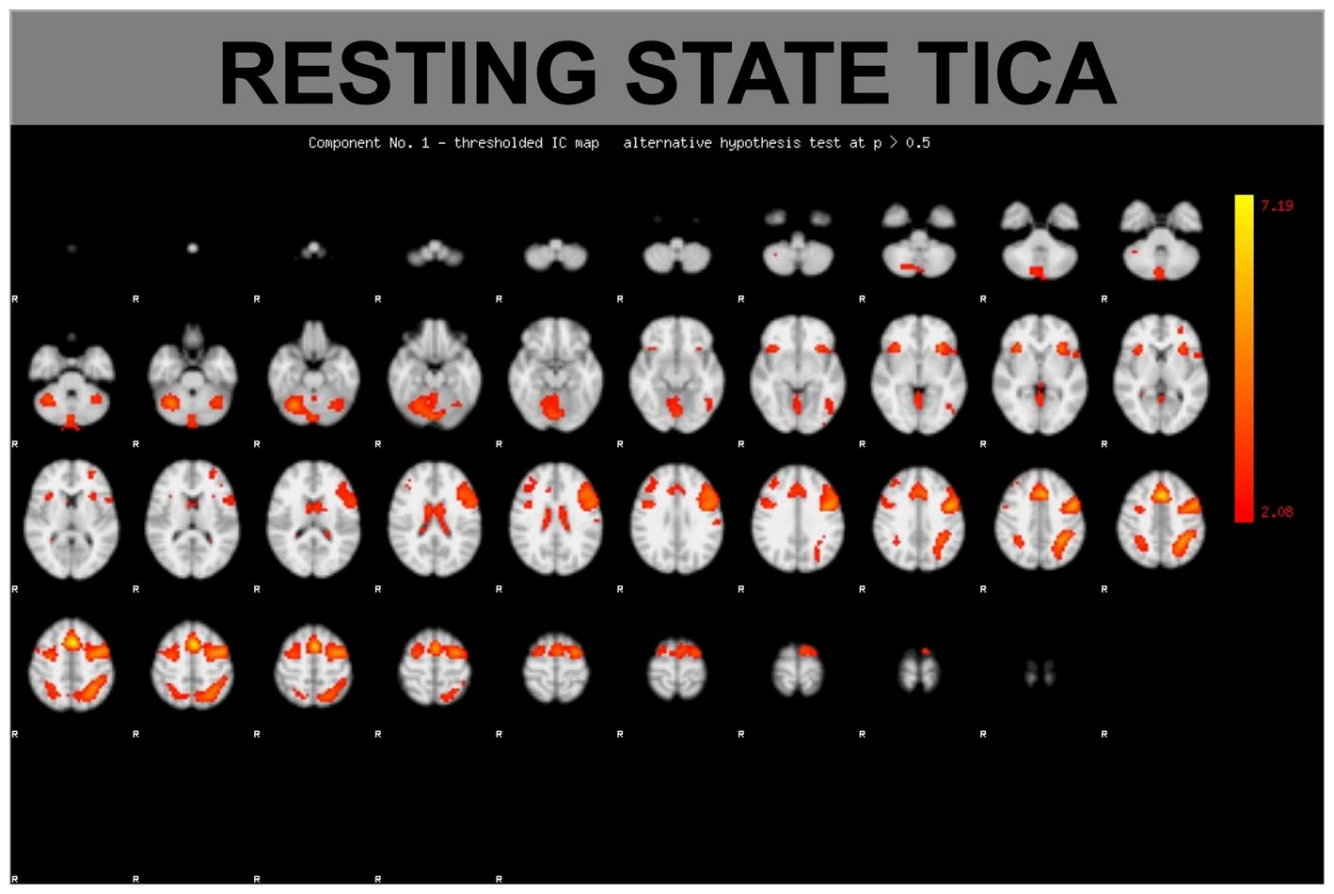
Example of a functional connectivity component based on a TICA (tensorial independent component) analysis.

The TICA resting fMRI analysis of both conditions MASK and NOMASK resulted in typical resting state networks (RSNs) (Figure 3). The direct comparison of the s-modes (a parameter of activation strength of those RSNs) for MASK versus NOMASK yielded no supra-threshold activations.

Moreover, the dual regression yielded no supra-threshold differences for MASK versus NOMASK (or inverse).

## 5 Discussion

The present study shows that wearing a facemask does not impact on both performances and brain activation patterns during a highly demanding 2-back working memory task. From a cognitive viewpoint, this finding is in line with several observations under normal conditions, high temperatures or mild exercise that demonstrated that the wearing of facemask does not or affect minimally the cognitive performances (Morris et al., 2020; Slimani et al., 2021; Wells et al., 2023). Only one study reported deficits in concentration and visual attention in the context of maximal running aerobic tests (Slimani et al., 2021). It is thus likely that, when wearing a facemask, a deleterious effect on cognition can be observed only under condition of motor or emotional stress.

Imaging data relative to the wearing of a facemask remain rare. In a series of 13 young individuals wearing a FFP2 facemask, Fischer et al., reported a 6.5% increase of cerebral blood flow and a 0.9% increase of oxygen saturation measured by transcranial hybrid near-infrared spectroscopy. The first fMRI study available in this field assessed the effect of wearing a facemask on functional MRI focusing on a basic sensory-motor task designed to activate visual, auditory, and sensorimotor cortices in eight middle-aged participants (Law et al., 2021). The authors reported no significant impact of facemask on task-related activation of sensorimotor areas. More recently, we analyzed the functional connectivity in a resting-state fMRI analysis of an independent sample of 23 healthy controls (Haller et al., 2022). This study reported no significant effect of wearing facemask on the functional connectivity of lower-level sensorimotor or visual networks but found a subtle impact on the interaction between the salience network as the seed region and the left middle frontal and precentral gyrus. More recently, Wu et al. examined the amplitude of low frequency fluctuation (ALFF) and functional connectivity at rest in 15 middle-aged healthy subjects wearing a KN95 mask and natural breathing. In contrast to the previous observations, they reported significant increases and decreases of ALFF as well as significant alterations of functional connectivity of posterior cingulate and medial prefrontal areas when wearing masks (Wu et al., 2023). These first fMRI studies in this field were performed with no or no significant cognitive challenge so that it was not possible to comment on a possible deleterious effect of the facemask in highly demanding situations. Because of its implication in numerous cognitive and cognitive-motor tasks, working memory is called upon in a wide range of activities. We decided to focus our analysis on this cognitive function since it is of key importance in daily life interactions and its brain correlates were very well established across the lifespan (Maillet and Rajah, 2014; Andre et al., 2016). Our findings indicate that wearing of the facemask does not change the functional connectivity patterns even when the demand of cognitive resources is high. These observations parallel the preliminary data of Klugah-Brown and collaborators (Klugah-Brown et al., 2022) who examined the effect of simple surgical mask on fMRI activation patterns during finger tapping, emotional face matching, working memory tasks with negative conclusions.

Some limitations should be considered when interpreting the present findings. To avoid the well-documented gender-related differences in functional connectivity (Murray et al., 2021), female participants were not included in this study. Moreover, we deliberately used a tight FFP2/KN95 facemask that has been the standard of reference the COVID-19 pandemic. It is, however, highly unlikely that the wearing of the less tight surgical facemasks led to significant changes in brain activation patterns. Last but not least, one should keep in mind that the 2-back task is a classical paradigm of working memory that involves attention and executive components but not emotional processing. This latter seems to be the most vulnerable domain of human cognition when wearing a facemask (Ferrari et al., 2021; Grundmann et al., 2021; Marini et al., 2021, 2022; Proverbio and Cerri, 2022; Tsantani et al., 2022; Proverbio et al., 2023). Future fMRI studies including tasks of social cognition are warranted to explore whether the wearing of facemask modifies brain activation patterns when dealing with emotional processing in complex environments.

## Data availability statement

The raw data supporting the conclusions of this article will be made available by the authors, without undue reservation.

## Ethics statement

The studies involving humans were approved by University of Geneva, Switzerland. The studies were conducted in accordance with the local legislation and institutional requirements. The participants provided their written informed consent to participate in this study. Written informed consent was obtained from the individual(s) for the publication of any potentially identifiable images or data included in this article.

## Author contributions

M-LM: Writing – review & editing, Writing – original draft, Visualization, Validation, Supervision, Software, Resources, Project administration, Methodology, Investigation, Funding acquisition, Formal Analysis, Data curation, Conceptualization. SH: Writing – review & editing, Writing – original draft, Visualization, Validation, Supervision, Software, Resources, Methodology, Investigation, Formal Analysis, Data curation, Conceptualization. CR: Writing – review & editing, Writing – original draft, Project administration. FH: Writing – review & editing, Writing – original draft, Formal Analysis. PG: Writing – review & editing, Writing – original draft, Visualization, Validation, Supervision, Software, Resources, Project administration, Methodology, Investigation, Funding acquisition, Formal Analysis, Data curation, Conceptualization.

## Footnotes

^1^ https://www.campusbiotech.ch/

^2^ https://www.graphpad.com
